# CD20 expression: A risk stratification factor for newly diagnosed multiple myeloma with t(11;14)

**DOI:** 10.3389/fonc.2022.1061438

**Published:** 2022-12-01

**Authors:** Yuan Jian, Zhiyao Zhang, Huixing Zhou, Guangzhong Yang, Chuanying Geng, Huijuan Wang, Wen Gao, Wenming Chen

**Affiliations:** Department of Hematology, Myeloma Research Center of Beijing, Beijing Chaoyang Hospital, Capital Medical University, Beijing, China

**Keywords:** multiple myeloma, CD20, t(11;14), prognosis, survival

## Abstract

**Objective:**

Translocation (11;14) is one of the most frequent recurrent cytogenetic abnormalities in multiple myeloma (MM), while its clinical prognostic value remains controversial. CD20 expression is uncommon in MM while strongly associated with t(11;14). This study aimed to investigate whether CD20 could provide further prognostic value in MM patients harboring t(11;14).

**Methods:**

CD20 expression detected by flow cytometry was retrospectively analyzed in 211 newly diagnosed MM patients with t(11;14). The clinical characteristics and outcomes were analyzed between CD20 positive and negative patients.

**Results:**

CD20 expression was found in 34.6% (73/211) newly diagnosed MM (NDMM) patients with t(11;14), associated with lower serum creatine levels and lower incidence of plasmacytoma. Based on similar treatment regimens, CD20 positive patients had a comparable overall response rate to CD20 negative patients, whereas had a lower CR/sCR (complete response/stringent complete response) rate than the latter (31.4% vs. 46.4%, P =0.045). Nevertheless, CD20 positive patients had a longer tendency of progression-free survival (PFS) (59.0 vs. 29.0 months, P =0.163) and significantly longer overall survival (OS) (99.0 vs. 56.0 months, P=0.003) than CD20 negative patients. Further investigation among CD20 expression proportion showed that strong expression of CD20 (>80% of bone marrow plasma cells) exhibited the longest OS (median not reached, P =0.011). However, the favorable impact of CD20 expression on survival was eliminated with the contaminant presence of cytogenetic abnormalities besides t(11;14). Autologous stem cell transplantation (ASCT) could improve the prognosis of CD20 negative t(11;14) patients. Multivariate analysis confirmed that CD20 expression was an independent favorable indicator for longer OS in t(11;14) MM patients.

**Conclusion:**

CD20 expression is a favorable prognostic factor in NDMM with t(11;14) and could provide further risk-stratification value in this heterogeneous disease subgroup.

## Introduction

Multiple myeloma (MM) is a plasma cell malignancy characterized with heterogeneous clinical presentation and outcomes. Among various prognostic factors described in MM, cytogenetic abnormalities (CAs) have a substantial impact on survival outcomes (1). Risk stratification according to CAs had provided a practicable prognostic system for MM patients (2, 3), however it still could not completely explain the huge discrepancy seen in different individuals. The chromosome translocation t(11;14)(q13;q32) is one of the most frequent recurrent CAs, with the prevalence of about 16% to 24% in newly diagnosed MM (NDMM) (4, 5). However, its clinical prognostic value remains controversial (4, 6, 7). Some patients harboring t(11;14) could stay alive for more than 10 years since diagnosis, while some could present as aggressive disease courses with short survival, indicating the t(11;14) in MM represents a heterogeneous subset with diverse cell origins and outcomes.

CD20 is a transmembrane phosphoprotein commonly expressed on different development stages of B lymphocytes (8). Although CD20 is not frequently expressed on plasma cells, reports had shown a prevalence of approximately 20% in NDMM cases (9), while strongly associated with t(11;14) translocation (10). However, researches on the prognostic role of CD20 in MM are quite limited while presenting with discrepancy results (7, 11, 12). Under this circumstance, we designed this study to investigate whether CD20 could provide further risk-stratification value in MM patients harboring t(11;14).

## Materials and methods

### Patients

A total of 211 NDMM patients diagnosed between July 2011 and January 2022 in Beijing Chaoyang Hospital (Beijing, China) with positive chromosome translocation of t(11;14) evaluated by interphase FISH (Fluorescence *in situ* hybridization) at diagnosis were retrospectively enrolled in this study. The diagnosis and staging of MM were according to the International Myeloma Working Group (IMWG) criteria (13). Among all the 211 patients, 187 were treated with bortezomib-based induction regimens (with or without lenalidomide), 2 were treated with lenalidomide-based regimens (without bortezomib), 3 were treated with daratumumab-based regimens and 19 with traditional chemotherapy regimens. Sixty-six patients had received autologous stem cell transplantation (ASCT). Response to therapy was evaluated based on IMWG criteria (14). The median follow-up duration was 52 (6-132) months by the cut-off date of 31 Jul 2022. This study has received approval from the Ethics Committee of Beijing Chaoyang Hospital. Written informed consents were obtained from all patients.

### FISH analysis

Bone marrow samples were purified with CD138 magnetic beads (Miltenyi Biotec, Germany) to obtain plasma cells and then were detected by specific probes to assess the following CAs: t(11;14), t(4;14), t(14;16), del(17p) and 1q21 gain. A total of at least 200 interphase nuclei were analyzed for each specimen. The thresholds were defined as the following: 10% for chromosome translocations including t(11;14), t(4;14) and t(14;16), while 20% for numerical abnormalities including del(17p) and 1q21 gains (15).

### Flow cytometry

Flow cytometric analysis was performed in all patients at diagnosis. Bone marrow cells were incubated and then detected with the following antibodies: CD19, CD20, CD45, CD56, CD138, CD269, cytoplasm IgM, cytoplasm κ and cytoplasm λ. Flow cytometry were analyzed by FACS DIVA software (BD Biosciences, San Jose, CA). An antigen expressed in more than 20% plasma cells was considered as positivity. Strong expression was defined as an antigen expressed in >80% of bone marrow plasma cells (BMPCs), while partial expression was defined as expressed in 20% to 80% of BMPCs.

### Statistical analysis

Statistical analyses were performed by SPSS version 24.0 (SPSS, Inc, Chicago, IL, USA). The *χ* ^2^ test or 2-sided Fisher exact test were used to compare categorical clinical characteristics. Wilcoxon rank-sum tests were used to compare continuous clinical characteristics. Progression-free survival (PFS) was defined as the time from the start of treatment to the first time of progression, or death from any cause. Overall survival (OS) was defined as the time from the start of treatment to death from any cause (16). Kaplan-Meier method was used to plot survival curves of PFS and OS, while log-rank test was employed to compare the differences between curves. Cox proportional hazard regression analysis was used to analyze the prognostic value of factors. *P*-value less than 0.05 was considered as statistical significance.

## Results

### Clinical characteristics of CD20 expression in MM patients with t(11;14)

Among all the 211 NDMM patients with t(11;14), CD20 expression was found in 34.6% (73/211) patients. Among the 73 patients with CD20 expression, 14 patients (19.2%) were strong expression, while the left (80.8%) were partial expression. Patient characteristics were summarized in **Table 1**. According to the results, t(11;14) patients presented a relatively high proportion of IgD (10.0%) and light chain (36.0%) type of M component, and also were more likely to be accompanied by peripheral blood plasma cells (16.8%), plasmacytoma (19.9%) and amyloidosis (8.1%). In the comparison according to CD20 expression, CD20 negative patients were presented higher serum creatine level (91.4 vs 77.6μmol/L, P =0.044) and higher incidence of plasmacytoma (23.9% vs 12.3%, P =0.048) than CD20 positive patients. No statistical differences were shown in the aspect of gender, disease stages, accompanied cytogenetics, hemoglobin, calcium, lactate dehydrogenase (LDH), albumin, β2-microglobulin and amyloidosis between the two groups (**Table 1**).

**Table 1:** Correlation between CD20 expression and clinical characteristics in MM patients with t(11;14).

	All patients (n=211)	CD20 positive (n=73)	CD20 negative (n=138)	*P* value
Gender				0.660
Male	126/211 (59.7)	42/73 (57.5)	84/138 (60.9)	
Female	85/211 (40.3)	31/73 (42.5)	54/138 (39.1)	
Age (years)	59 (28-86)	59 (28-84)	59 (29-86)	0.240
DS stage				0.492
I	6/208 (2.9)	2/71 (2.8)	4/137 (2.9)	
II	32/208 (15.4)	8/71 (11.3)	24/137 (17.5)	
III	170/208 (81.7)	61/71 (85.9)	109/137 (79.6)	
ISS stage				0.310
I	41/209 (19.6)	15/71 (21.1)	26/138 (18.8)	
II	59/209 (28.2)	24/71 (33.8)	35/138 (25.4)	
III	109/209 (52.2)	32/71 (45.1)	77/138 (55.8)	
R-ISS stage				0.490
I	37/206 (18.0)	15/70 (21.4)	22/136 (16.2)	
II	139/206 (67.5)	47/70 (67.1)	92/136 (67.6)	
III	30/206 (14.6)	8/70 (11.4)	22/136 (16.2)	
M component				0.658
IgG	74/211 (35.1)	27/73 (37.0)	47/138 (34.1)	
IgA	27/211 (12.8)	10/73 (13.7)	17/138 (12.3)	
IgD	21/211 (10.0)	8/73 (11.0)	13/138 (9.4)	
Light chain	76/211 (36.0)	26/73 (35.6)	50/138 (36.2)	
Nonsecretory	13/211 (6.2)	2/73 (2.7)	11/138 (8.0)	
Light chain type				0.311
κ	103/211 (48.8)	36/73 (49.3)	67/138 (48.6)	
λ	95/211 (45.0)	35/73 (47.9)	60/138 (43.5)	
Others	13/211 (6.2)	2/73 (2.7)	11/138 (8.0)	
Accompanied cytogenetics				
17p deletion	13/211 (6.2)	3/73 (4.1)	10/138 (7.2)	0.549
t(4;14)	4/211 (1.9)	1/73 (1.4)	3/138 (2.2)	1.000
t(14;16)	2/211 (0.9)	0/73 (0.0)	2/138 (1.4)	0.545
1q21 amplification	82/211 (38.9)	26/73 (35.6)	56/138 (40.6)	0.553
Hemoglobin (g/L)	95 (46-156)	92 (51-142)	97 (46-156)	0.098
Calcium (mmol/L)	2.31 (1.47-3.95)	2.28 (1.48-3.25)	2.33 (1.47-3.95)	0.385
Lactate dehydrogenase (U/L)	167 (20-492)	162 (20-492)	170 (69-487)	0.082
Serum creatinine (μmol/L)	85.0 (36.7-1436.6)	77.6 (36.7-736.2)	91.4 (43.4-1436.6)	0.044*
Albumin (g/L)	36.1 (12.5-51.2)	35.7 (18.8-46.7)	37.0 (12.5-51.2)	0.114
β2-microglobulin (mg/L)	5.65 (1.00-67.60)	4.98 (1.85-67.60)	5.98 (1.00-61.60)	0.337
Amyloidosis	17/211 (8.1)	6/73 (8.2)	11/138 (8.0)	1.000
Plasmacytoma	42/211 (19.9)	9/73 (12.3)	33/138 (23.9)	0.048*
Peripheral blood plasma cells	33/196 (16.8)	8/66 (12.1)	25/130 (19.2)	0.232
Bortezomib-based induction regimens	187/211 (88.6)	64/73 (87.7)	123/138 (89.1)	0.821
ASCT^#^	66/211 (31.3)	26/73 (35.6)	40/138 (29.0)	0.323

Data are presented as n (%) or median (range).

# ASCT, autologous stem cell transplantation.

* means P<0.05.

### CD20 expression and response rate

Among all 211 patients, 187 (88.6%) had received bortezomib-based induction therapy, while 66 (31.3%) had received ASCT. The proportion of patients receiving bortezomib-based induction regimens (87.7% vs. 89.1%, P =0.821) and ASCT (35.6% vs. 29.0%, P =0.323) were similar between the CD20 positive and CD20 negative groups (**Table 1**). A total of 182 patients had evaluable efficacy to first-line therapy. The correlation of CD20 expression and best response rates was analyzed (**Table 2**). With the results, the overall response rates (ORR) were comparable between CD20 positive and negative groups (90.0% vs. 84.8%, P =0.315). However, CD20 negative group had a higher CR/sCR (complete response/stringent complete response) rate than CD20 positive group (46.4% vs. 31.4%, P =0.045). These results showed that t(11;14) patients with positive CD20 expression were less likely to reach deeper responses while had similar ORRs compared to those without CD20 expression.

**Table 2:** Response rate according to CD20 expression.

Response	CD20 positive (n=70)	CD20 negative (n=112)
sCR	14/70 (20.0)	22/112 (19.6)
CR	8/70 (11.4)	30/112 (26.8)
VGPR	22/70 (31.4)	18/112 (16.1)
PR	19/70 (27.1)	25/112 (22.3)
SD/PD	7/70 (10.0)	17/112 (15.2)

sCR, stringent complete response; CR, complete response; VGPR, very good partial response; PR, partial response; SD, stable disease; PD, progressive disease.

Data are presented as n (%).

### Survival analysis

**Figure 1:**
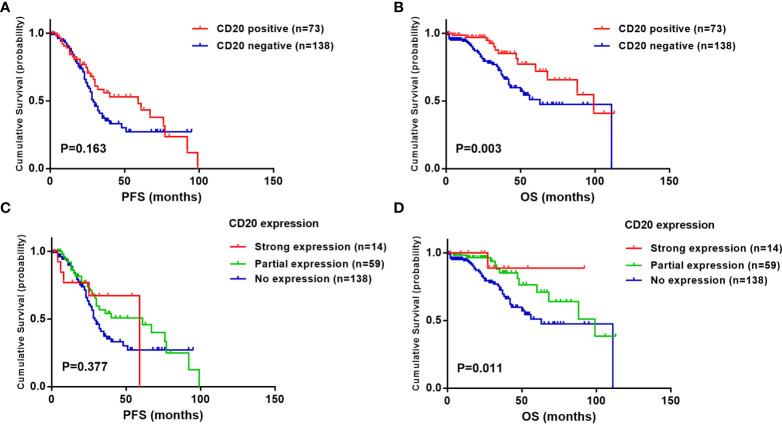
Effect of CD20 expression on survival in t(11;14) MM patients. **(A, B)** PFS and OS in relation to CD20 expression. **(C, D)** PFS and OS in relation to CD20 expression proportion. Strong expression: CD20 expressed in >80% of bone marrow plasma cells (BMPCs); partial expression: CD20 expressed in 20% to 80% of BMPCs; no expression: CD20 expressed in less than 20% of BMPCs.

In the whole cohort of t(11;14) patients, the median PFS was 32.0 months, and the median OS was 88.0 months. These survival times were further evaluated according to CD20 expression. With the results, CD20 positive patients had a longer tendency of PFS than CD20 negative patients (59.0 vs. 29.0 months, P =0.163). However the survival advantage on PFS did not reach statistical significance (**Figure 1A**). In the aspect of OS, CD20 positive patients had significantly longer overall survival than CD20 negative patients. The median OS in CD20 positive patients was 99.0 (95% CI: 61.2-136.8) months, while in those without CD20 expression was 56.0 (95% CI: 40.5-71.5) months (P =0.003) (**Figure 1B**).

The relevance between CD20 expression proportion and survival time was further investigated. Despite no significant difference shown in PFS (**Figure 1C**), results revealed that patients with strong expression of CD20 (>80% of bone marrow plasma cells) exhibited the longest overall survival time (P =0.011), with the median OS still not reached. Survival time in patients with partial CD20 expression (20%-80% of BMPCs) was longer than those without CD20 expression (median OS: 99.0 vs. 56.0 months), while still shorter than strong expression population nevertheless (**Figure 1D**).

**Figure 2:**
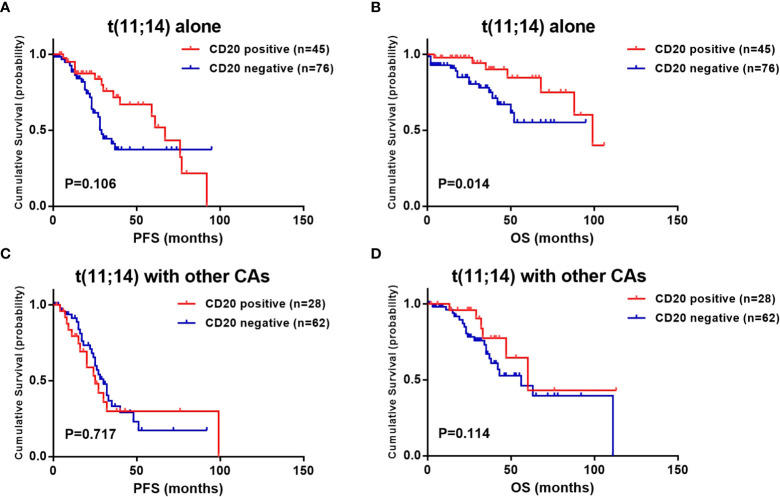
Effect of CD20 expression on survival in t(11;14) MM patients based on accompanied cytogenetic abnormalities (CAs) [t(4;14), t(14;16), del(17p) and/or 1q21 gain]. **(A, B)** PFS and OS in relation to CD20 expression in patients with t(11;14) alone. **(C, D)** PFS and OS in relation to CD20 expression in patients with t(11;14) and other accompanied CAs.

In our patient cohort, a total of 4 cytogenetic abnormalities were detected beside t(11;14), including t(4;14), t(14;16), del(17p) and/or 1q21 gain. Of all 211 patients, 2 patients (0.9%) carried 3 additional CAs, 7 (3.3%) carried 2 additional CAs, 81 (38.4%) carried 1 additional CA, while the other 121 patients (57.3%) had no additional CAs. The effect of accompanied CAs on survival was subsequently analyzed. With the results, CD20 expression exhibited its survival advantage specifically on those with t(11;14) alone (median PFS: 67.0 vs. 29.0 months, P =0.106; 5-year OS: 84.5% vs. 53.1%, P =0.014) (**Figures 2A, B**). When other accompanied CAs presented, there were no significant differences in survival between CD20 positive and negative patients (median PFS: 25.0 vs. 30.0 months, P =0.717; median OS: 60.0 vs. 56.0 months, P =0.114) (**Figures 2C, D**).

**Figure 3:**
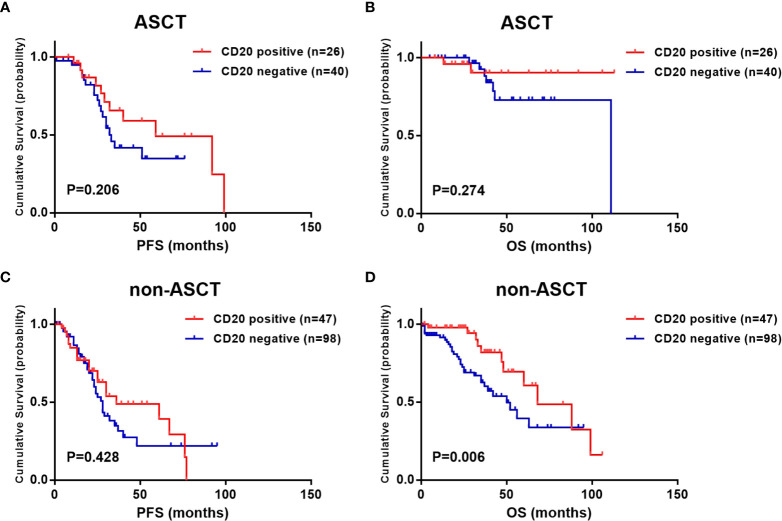
Effect of CD20 expression on survival in t(11;14) MM patients based on different treatments. **(A, B)** PFS and OS in relation to CD20 expression in ASCT patients. **(C, D)** PFS and OS in relation to CD20 expression in non-ASCT patients.

To reduce the effect of the heterogeneity of treatment regimen on survival analysis, we further analyzed the prognostic value of CD20 expression in ASCT and non-ASCT patients separately. According to the results, the survival advantage of CD20 expression was attenuated by ASCT. PFS and OS were comparable in CD20 positive and negative patients who received ASCT (median PFS: 59.0 vs. 33.0 months, P =0.206; median OS: not reached vs. 111.0 months, P =0.274) (**Figures 3A, B**). While in those who did not receive ASCT, although the PFS was not shown differences (36.0 vs. 28.0 months, P =0.428), CD20 positive patients still exhibited longer OS than CD20 negative patients (68.0 vs. 50.0 months, P =0.006) (**Figures 3C, D**).

### Multivariate analysis

Univariate and multivariate analyses were performed to explore the influence of CD20 expression and relevant clinical parameters on OS. According to the univariate analyses, age ≥65 years, R-ISS stage III, calcium ≥2.6mmol/L, serum creatinine ≥130μmol/L, LDH ≥250U/L, high-risk cytogenetics and peripheral blood plasma cells were associated with shorter OS, while CD20 expression was associated with longer OS (**Table 3**). In multivariate analysis, the prognostic values of these parameters were further examined. With the results, CD20 expression retained its favorable impact on OS (HR 0.600, 95% CI: 0.363-0.994, P =0.047). Other factors that were significant in multivariate analysis included age ≥65 years (95% CI: 1.170-3.959, P =0.014) and high-risk cytogenetics (95% CI: 1.485-5.888, P =0.002), both of which indicating shorter OS (**Table 4**).

**Table 3:** Univariate analysis of variables associated with outcomes in MM patients with t(11;14).

Parameter	OS		
	HR	95% CI	*P* value
Age (years) ≥ 65 vs. < 65	1.756	1.004-3.072	0.048*
R-ISS stage III vs. I-II	2.268	1.212-4.245	0.010*
Calcium (mmol/L) ≥ 2.6 vs. < 2.6	2.448	1.401-4.278	0.002**
Serum creatinine (μmol/L) ≥ 130 vs. < 130	2.579	1.517-4.384	<0.001***
Hemoglobin (g/L) < 90 vs. ≥ 90	1.656	0.973-2.820	0.063
Lactate dehydrogenase (U/L) ≥ 250 vs. < 250	2.076	1.036-4.162	0.039*
High-risk cytogenetics^#^ Positive vs. negative	2.957	1.485-5.888	0.002**
1q21 amplification Positive vs. negative	1.090	0.640-1.857	0.752
CD20 expression Positive vs. negative	0.402	0.215-0.752	0.004**
Amyloidosis Yes vs. no	1.189	0.509-2.782	0.689
Plasmacytoma Yes vs. no	1.089	0.547-2.165	0.809
Peripheral blood plasma cells Yes vs. no	2.978	1.492-5.941	0.002**

HR, harzard ratio; CI, confidence interval.

# High-risk cytogenetics: del(17p), t(4;14) and/or t(14;16).

* means p<0.05, ** means p<0.01, *** means p<0.001.

**Table 4:** Multivariate analysis of variables associated with outcomes in MM patients with t(11;14).

Parameter	OS		
	HR	95% CI	*P* value
Age (years) ≥ 65 vs. < 65	2.153	1.170-3.959	0.014*
R-ISS stage III vs. I-II	0.622	0.201-1.926	0.410
Calcium (mmol/L) ≥ 2.6 vs. < 2.6	1.871	0.994-3.523	0.052
Serum creatinine (μmol/L) ≥ 130 vs. < 130	1.724	0.923-3.217	0.087
Lactate dehydrogenase (U/L) ≥ 250 vs. < 250	1.695	0.555-5.178	0.355
High-risk cytogenetics^#^ Positive vs. negative	2.957	1.485-5.888	0.002**
CD20 expression Positive vs. negative	0.600	0.363-0.994	0.047*
Peripheral blood plasma cells Yes vs. no	2.007	0.958-4.204	0.065

HR, harzard ratio; CI, confidence interval.

# High-risk cytogenetics: del(17p), t(4;14) and/or t(14;16).

* means p<0.05, ** means p<0.01.

## Discussion

The t(11;14) multiple myeloma has been surrounded by many controversies, including its indefinite biological characteristics, distinct clinicopathological features, especially its inconclusive prognostic values (11). Some studies showed that patients with t(11;14) should be considered as standard-risk (4, 7, 17), while other argued that t(11;14) was a negative indicator associated with inferior survival to other standard-risk patients, especially in novel agent era (6, 18, 19). In general, t(11;14) was recognized as a group of heterogeneous disease, which had shown huge discrepancies in outcomes (11, 20, 21). Herein we obtained credible data from 211 newly diagnosed MM patients with t(11;14) and analyzed whether CD20 expression detected by flow cytometry could further separate the different prognosis outcomes of t(11;14). With our results, patients with or without CD20 expression exhibited different clinical characteristics. CD20 positive patients showed superiority in much longer survival and better outcome.

As usually expressed on the surface of B lymphocytes, CD20 still could be seen in 7.5%-22% of newly diagnosed MM patients (9, 22). Within common cytogenetic abnormalities in MM, CD20 expression was most frequently seen in t(11;14), with a prevalence rate of about 40-45% (12, 23), which is significantly higher than those without t(11;14) with the incidence was only 4.5% (23). According to our results, CD20 could be identified in 34.6% of all 211 patients with t(11;14), which was in accordance with previously published studies (12, 23). CD20 expression did not show specific correlations with the typical characteristics of MM such as disease type, stage and accompanied high-risk cytogenetics. Peripheral blood plasma cells were more frequently seen in the whole t(11;14) cohort (16.8%), while did not show a statistical difference between the groups. However, CD20 negative patients presented with impaired renal function and a high incidence of plasmacytoma, both of which could contribute to inferior outcomes of MM (24, 25).

It was also controversial that whether CD20 expression conferred a good or poor prognostic value in MM (23, 26). According to some previous studies, the prognostic value of CD20 varies among different disease subtypes. An et al. (11) showed a good prognosis of CD20 expression in t(11;14) MM, while Huang et al. (12) demonstrated both a longer PFS and OS of CD20 expression in non-IgD MM. Another report stated that CD20 positive MM was a group of heterogeneous diseases, of which CCND1 displayed good prognosis while CCND2 displayed aggressive disease with a poor prognosis (27). In the present study, a favorable prognostic value of CD20 on overall survival was revealed in NDMM patients harboring t(11;14), indicating CD20 expression could provide additional prognostic information in this specific t(11;14) subgroup.

The prognosis of t(11;14) patients would be strongly affected by co-existing CAs. A previous study reported that t(11;14) with additional CAs such as 1q gain, del(1p), del(IGH), del(17p) and del(13q) had exerted a deleterious effect on median OS compared with t(11;14) alone (28). A recent study also showed that the coexistence of del(17p) and t(11;14) presented with high-risk characteristics with high early death risk and short survival (21). As to chromosomal abnormalities detected by G-banding, it has also been reported that t(11;14) group with additional chromosomal abnormalities (ACAs) had significantly shorter survival than those without ACAs, and even than t(4;14) group in MM patients receiving ASCT (29). However, our previous study showed that the contaminant presence of 1q21 gain did not confer a worse prognosis than t(11;14) alone in ASCT patients (7). Thus, the prognosis value of CD20 in patients with contaminant CAs needs to be further discussed. In the present study, we found that CD20 lost its prognostic value when other CAs coexisted with t(11;14), indicating that the contaminant CAs had changed the biological characteristics of t(11;14).

An interesting finding in our results that needs to be noted is, with similar therapeutic regimens, CD20 positive patients were less likely to reach deep response (CR/sCR) than CD20 negative patients, whereas exhibited longer survival than the latter. In a previous study, Huang et al. (12) also reported a slow onset of response while comparable best response and longer survival of CD20 positive MM patients. Considering the fact that patients with t(11;14) usually exhibited a decreased proliferative index and a lower deep response rate (20, 30), it could be speculated that CD20 positive t(11;14) myeloma referred to a more indolent clinical course with a better outcome.

One aspect of the heterogeneity of MM manifested as, even in the same patient, not all plasma cells expressed CD20. In our t(11;14) cohort, among the 73 patients with CD20 expression, only 19.2% were strong expression (CD20 expressed in more than 80% of BMPCs), while in the others, CD20 expressed only in parts of BMPCs (20% to 80%). In the survival analysis, results showed a strong correlation between CD20 expression proportion and survival time. Patients with strong expression of CD20 exhibited the longest OS (median not reached). This correlation further confirmed the favorable prognostic impact of CD20 expression on t(11;14) patients.

The best treatment strategies on t(11;14) MM need to be further explored according to CD20 expression. With our results, the survival advantage of CD20 expression had been attenuated by ASCT. This finding was in accordance with a previous study from our team, which demonstrated that CD20 expression did not show a significant prognosis impact among MM patients with t(11;14) alone while receiving ASCT (7), indicating that ASCT could improve the poor prognosis in CD20 negative t(11;14) patients. However, a retrospective study showed that in CD20 positive patients, novel agents and ASCT did not show significant survival benefits (12). In consideration of the small study population and the retrospective property of the existing studies, the impact of ASCT on CD20 expression in such patients still needs to be further clarified.

Since CD20 positive t(11;14) myeloma presents more like an indolent disease, it might be possible to combine anti-CD20 monoclonal antibody with conventional anti-MM therapy to further improve response rate and eliminate residual disease (31), and to further improve survival of this MM subgroup that have already been with good prognosis, probably could turn this group of patients into a subgroup with the best prognosis in MM. However, considering limited clinical data, this point of view remains needing to be further explored. The Bcl-2 inhibitor venetoclax has also exhibited inspiring efficacy in t(11;14) MM patients (32, 33). As the relationship between Bcl-2 and CD20 expression still need to be clarified, it remains to be discussed to develop targeted therapies in such patients, and the prognostic relevance of CD20 expression under targeted therapies.

There are also some limitations in this study. As a retrospective study, one of the main limitations is the heterogeneity of treatment regimens, including induction, consolidation and maintenance regimens, resulting in difficulty to interpreting the impact of different therapy on survival. Another limitation is that repeating detections of CD20 and t(11;14) at MM relapse were not available in most patients, so it is still unknown whether CD20 loss after treatment would contribute to a worse outcome. Larger prospective studies and continuous monitoring of CD20 are needed to further clarify the prognostic role of CD20 in t(11;14) MM patients.

In conclusion, our findings suggest that CD20 expression could provide important prognostic value in NDMM patients harboring t(11;14) and should be considered as an important additional risk stratification factor in these specific patients.

## Data availability statement

The original contributions presented in the study are included in the article/supplementary materials. Further inquiries can be directed to the corresponding author.

## Ethics statement

The studies involving human participants were reviewed and approved by Ethics Committee of Beijing Chaoyang Hospital. The patients/participants provided their written informed consent to participate in this study.

## Author contributions

WG and WC designed this study. YJ, HZ, GY, CG, and HW provided study patients and collected data. YJ, HZ, and ZZ performed data analysis. YJ and HZ wrote the manuscript. WC supervised the study. All authors contributed to the article and approved the submitted version.

## Funding

This project was supported by Training Fund for Open Projects at Clinical Institutes and Departments of Capital Medical University (CCMU2022ZKYXY014).

## Conflict of interest

The authors declare that the research was conducted in the absence of any commercial or financial relationships that could be construed as a potential conflict of interest.

## Publisher’s note

All claims expressed in this article are solely those of the authors and do not necessarily represent those of their affiliated organizations, or those of the publisher, the editors and the reviewers. Any product that may be evaluated in this article, or claim that may be made by its manufacturer, is not guaranteed or endorsed by the publisher.
